# Litter Survival Differences between Divergently Selected Lines for Environmental Sensitivity in Rabbits

**DOI:** 10.3390/ani9090603

**Published:** 2019-08-25

**Authors:** Ivan Agea, María-Luz García, Agustín Blasco, María-José Argente

**Affiliations:** 1 Departamento de Tecnología Agroalimentaria. Universidad Miguel Hernández de Elche, Ctra de Beniel Km 3.2, 03312 Orihuela, Spain; 2 Institute for Animal Science and Technology, Universitat Politècnica de València, P.O. Box 22012. 46022 Valencia, Spain

**Keywords:** correlated response, pre-weaning, survival, weight, welfare

## Abstract

**Simple Summary:**

Two rabbit lines are divergently selected for increasing or decreasing the variability of litter size at birth. Decreasing the litter size variability produces more resilient females with less sensitivity to diseases, being an indirect selection way to improve environmental sensitivity. The kits’ survival rate at weaning was higher in the homogeneous line. Moreover, this line led to a greater uniformity of the kits’ weight at weaning, although the weight variability at birth was higher, which could be due to a higher lactation capacity of the homogeneous line.

**Abstract:**

A divergent selection experiment on environmental sensitivity was performed in rabbits. The aim was to estimate the correlated response in kit weight and survival, litter weight, and weight distance from birth to weaning. The weight distance was calculated as the absolute value of the differences between the individual value and the mean value of its litter. The relationship between the probability of survival at 4 d of age, and the weight at birth, was studied. Environmental sensitivity was measured as litter size variability. A total of 2484 kits from 127 does from the low line, and 1916 kits of 114 does from the high line of the 12th generation were weighed. Both of the lines showed similar individual and litter weights at birth and weaning, and a similar survival rate at birth, and at 4 d of age. The survival rate at weaning was higher in the low line (0.67 and 0.62; P = 0.93). The weight distance was higher at birth, but lower at weaning in the low line (47.8 g and 54.1 g; P = 0.98). When the weight at birth was high, the kits had a higher survival rate. In conclusion, selection for environmental sensitivity showed a correlated response in the kits’ survival, and in the homogeneity of litter weight at weaning.

## 1. Introduction

The aim of genetic selection in maternal rabbit lines has traditionally been to improve the mean of productive traits: Litter size [1], or the length of does’ productive life [2,3]. Overall, this intensive selection for the increase of productivity has been successful, but it has also had negative consequences on animal welfare, increasing culling at early ages [4,5]. Consequently, resistance toward disease and stress are current priorities in rabbit breeding, leading to better doe resilience and welfare.

Selection for environmental sensitivity, measured as litter size variability, is an indirect selection methodology for improving resilience and robustness [6,7]. A divergent selection experiment for this trait has been performed with success [6], leading to lines with high and low litter size variability. Higher litter size variability affects the heterogeneity of littermates, which can produce lower pre-weaning survival rates [8,9]. The aim of this work is to study the correlated response in the pre-weaning survival rates of two rabbit lines, divergently selected for environmental sensitivity.

## 2. Material and Methods

All experimental procedures involving animals were approved by the Miguel Hernández University of Elche Research Ethics Committee (Reference number 2019/VSC/PEA/0017), in accordance with Council Directives 98/58/EC and 2010/63/EU.

### 2.1. Animals

A divergent selection experiment for litter size variability was carried out over twelve generations. The selection was based on the phenotypic variance of the litter size of each doe, after correcting the litter size for both year–season and parity–lactation status [6].

All of the animals were reared in the farm of the Miguel Hernández University of Elche (Spain). The rabbits were fed a standard commercial diet (17% crude protein, 16% fiber, 3.5% fat, Nutricun Elite Gra^®^, De Heus Nutrición Animal, La Coruña, Spain). Food and water were provided ad libitum. The same feeding conditions were provided for both lactating and non-lactating does. Does were housed in individual cages (37.5 × 33 × 90 cm) under a constant photoperiod of 16 h continuous light: 8 h continuous darkness, and with controlled ventilation throughout the experiment. The experiment took place from December to September. The temperature and relative humidity were recorded every 15 min with a Tinytag data logger (Table 1).

Does were first mated at 18 weeks of age, and at 10 d after parturition thereafter. Matings took place every week. The nest was made with textile by-products and the doe had free access to the nest, from 2 days before delivery until 21 days after delivery, when the nest was removed. The litters were not standardized, and the kits were weaned at 28 days of age.

Data come from the 12th generation of the selection. The litter size at birth (LS), the number born alive (NBA), the number born dead (NBD), the number of rabbits at 4 days of age (N4), and the number of rabbits at weaning (NW) were recorded. The rabbits were individually weighed and sexed within 24 h after birth. Some of the kits had suckled before being weighed. The milk intake was verified by recording a white mark in the abdominal area. The kits were also weighed at weaning. A total of 2484 kits from 127 does from the low line, and 1916 kits of 114 does from the high line were weighed.

### 2.2. Traits

The following traits were analyzed: LS; survival at birth (NBA/LS); survival at 4 days of age (N4/NBA); survival at weaning (NW/N4); the individual weight at birth of live and dead kits; the individual weight at weaning; the litter weight at birth of total kits and kits alive; the litter weight at weaning; and the weight distance of live, dead, and weaned rabbits. The weight distance was calculated as the absolute value of the differences between the individual value and the mean value of its litter.

### 2.3. Statistical Analysis

The model used for analyzing the LS and the litter survival rates was:

y_ijkl_ = µ + L_i_ +S_j_ + LP_k_ + p_ijkl_ + e_ijkl_

where L_i_ is the line effect with two levels (the high and the low lines); S_j_ is the season effect with three levels (winter, spring, and summer); LP_k_ is the lactation–parity effect with five levels (nulliparous, lactating, and non-lactating primiparous doe, and lactating and non-lactating multiparous doe); p_ijkl_ is the dam permanent effect with 241 levels; and e_ijkl_ is the residual term.

The individual weight at birth for the live and dead kits, and their corresponding distance were analyzed using the following model:

y_ijklmnop_ = LK_i_ + S_j_ + LP_k_ + IM_l_ + SE_m_ + p_ijklmn_ + c_ijklmno_ + b × LS_ijklmno +_ e_ijklmnop_

where LK_i_ is the line-survival effect (live kits of the high line, dead kits of the high line, live kits of the low line, and dead kits of the low line); IM_l_ is the intake of milk effect (whether the kit suckled or not before being weighed); SE_m_ is the sex effect (male and female); p_ijklmn_ is the dam permanent effect with 241 levels; c_ijklmno_ is the common litter effect with 541 levels; b is the regression coefficient of the covariate; LS_ijklmno_ is the covariate litter size; and e_ijklmnop_ is the residual term. 

Litter weights, individual weights at weaning, and the distance were analyzed with the same model, but the line effect with two levels (high and low lines) was used instead of the line-survival effect.

All of the analyses were performed using Bayesian methodology [10]. Bounded uniform priors were used for all effects. The joint prior distribution for the permanent environmental effect of the doe and the common litter effect was N (0, I⨂G_p_), where G_p_ was the (co)variance matrix between these effects. Residuals priori distribution was N (0, I⨂σ^2^_e_). Residuals, permanent environmental effects, and common litter effects are uncorrelated. The priors for the variances were also bounded uniform. Features of the marginal posterior distributions for all of the unknowns were estimated using Gibbs sampling. The Threshold Model program was used [11]. We used a chain of 250,000 samples, with a burn-in period of 50,000. Only one out of every 100 samples was saved for inferences. Convergence was tested using the Z criterion of Geweke [12], and Monte Carlo sampling errors were computed using time-series procedures [13].

The relationship between the probability of survival from birth to 4 d of age, and the individual weight at birth was analyzed by logistic regression. The model included line, season, parity–lactation (with three levels: Nulliparous, lactating, and non-lactating does), milk intake, and sex effects. Table 2 shows the number of kits that survived at 4 d of age, classified by weight at birth, and line. The LOGISTIC procedure of the statistical package SAS was used [14].

## 3. Results

### 3.1. Correlated Response to Selection in Litter Survival and Pre-Weaning Weight

Descriptive results of the traits by line are presented in Table 3. The coefficient of variations are moderate and increase from birth to weaning, except for the weight distance, which is high and similar.

Table 4 shows the features of the estimated marginal posterior distributions of the differences between the lines for litter survival, individual weight, and weight distances at birth and weaning. The litter size at birth was higher in the low line (H-L = −0.6 kits; P = 1.0). The survival rate at birth and at 4 d of age were similar between the lines, but the survival at weaning was 5% higher in the low line (P = 0.93). Both of the lines showed similar individual weights of kits, and litter weight at birth. There is some evidence that the individual weight at weaning was lower in the low line (H-L = 15 g; P = 0.82), but when the litter weight at weaning was considered, both lines showed similar values (P = 0.78). The weight distance for live kits at birth was higher in the low line (H-L = −0.5 g; P = 0.97); however, the weight distance at weaning was lower in the low line (H-L = 6.3 g; P = 0.98).

### 3.2. Survival at 4 d of Age and Individual Weight at Birth

**Figure 1:**
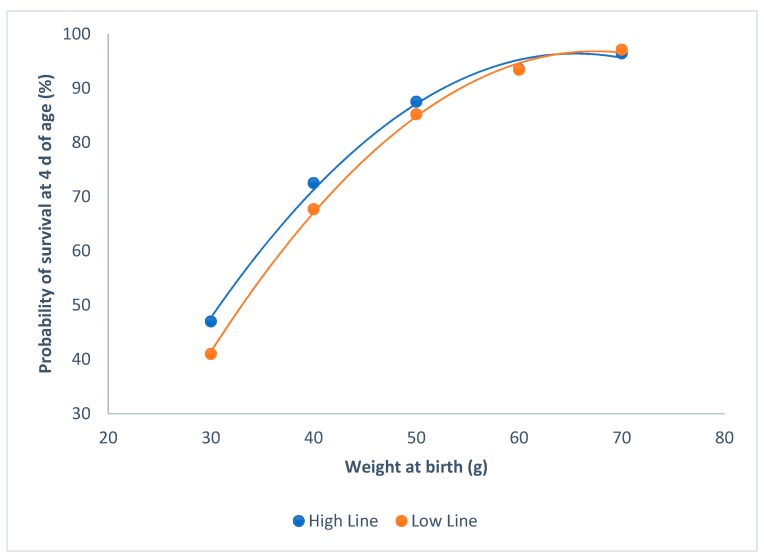
Relationship between survival at 4 d of age and individual birth weights for the high and the low litter size variability lines.

The probability of survival at 4 d of age, and weight at birth were not affected by sex (P = 0.47). Both of the lines showed similar probabilities of survival at 4 d of age, with the same weight at birth (P = 0.12; Figure 1). Probabilities of survival asymptotically increased with the individual birth weights, and raised to more than 90% from 60 g onwards.

**Figure 2:**
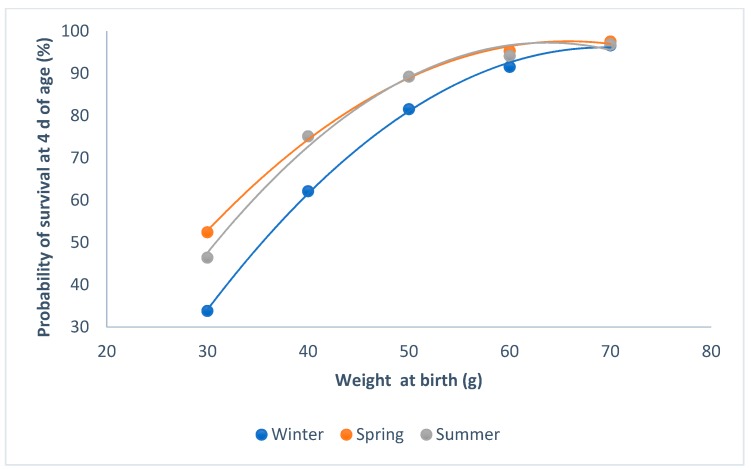
Relationship between survival at 4 d of age and individual birth weight for the seasons.

**Figure 3:**
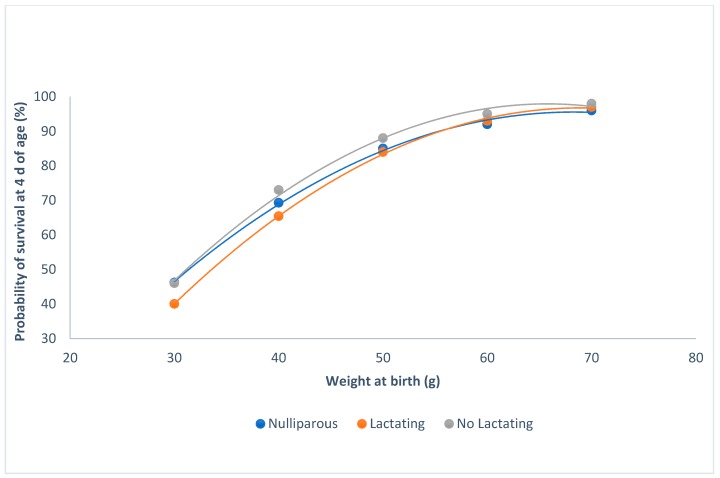
Relationship between survival at 4 d of age and individual birth weight for the parity-lactation status.

Kits born in winter had less of a probability of survival than those that were born in summer or spring (P < 0.0001; Figure 2). When the weight of the kits was higher than 60 g at birth, the probability of survival was at its maximum, regardless of the parity–lactation status of the doe (P < 0.0001; Figure 3). The minimum probability of survival took place in the lactating does, when the weights ranged from 30 to 60 g; the non-lactating does showed the highest probability of survival.

**Figure 4:**
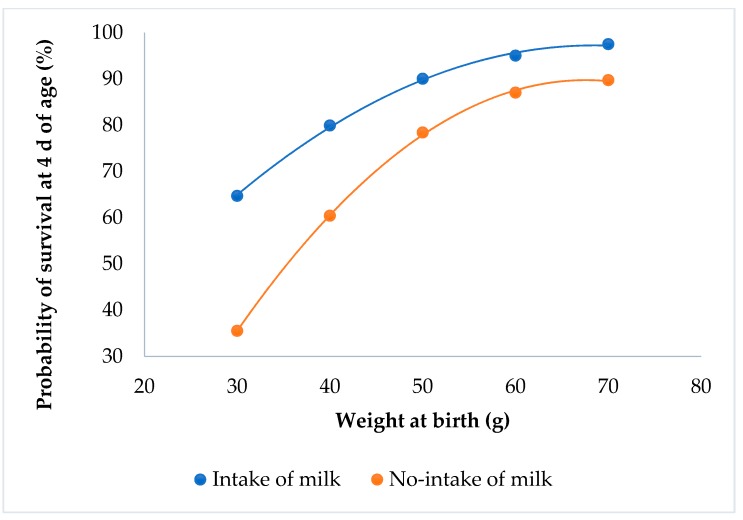
Relationship between survival at 4 d of age and individual birth weight for the milk intake effect.

Kits that suckled always had a higher probability of survival than the kits that did not suckle (P < 0.0001; Figure 4). Kits with the minimum weight had a survival probability of 65% when the rabbits suckled, but only 35% if they did not suckle.

## 4. Discussion

### 4.1. Correlated Response to Selection in Litter Survival and Pre-Weaning Weight

Our divergent selection experiment for environmental sensitivity has shown that this trait is genetically determined [6]. This has implications for animal welfare, as animals that cope better with their environment have better welfare than the more sensitive animals [7]. After correcting for the litter size, both of the lines had similar individual weights at birth, and the survival rates at birth and the survival rates at four days of age were not modified. Moreover, the relationship between the probability of survival at 4 d of age and the weight at birth was not affected by the line.

Weight distance has been used as the dispersion measure, instead of the standard deviation of the weight of the litter, because it provides one record per individual instead of one per litter. It seems that there is a correlated response on both of the weight distances at birth and at weaning, but with opposite sign; the kits’ weight is more variable at birth in the low line, but then less variable at weaning. To date, there is no information available on the weight distance at birth in rabbits, but Peiró et al. have shown similar values of weight distance at weaning [15].

Maternal care in the first days after parturition is clearly related to the ingestion of energy by the kits, which is directly related to their survival [16]. So, the higher rate of survival at weaning of the low line could indicate higher milk production, and better maternal behavior during lactation. In spite of the greater variability of weight at birth of the low line, this line produces a greater uniformity of weight at weaning than the high line, perhaps due to a higher lactation capacity of the doe. The homogeneity in weight within the litter is an important trait in prolific species such as rabbits [17], because increasing the weight homogeneity within the litter reduces the competition between littermates, and increases the viability of them [18].

### 4.2. Survival at 4 d of Age and Individual Weight at Birth

The probability of individual survival at 4 d of age is related to birth weight, as the kits with lower birth weight have a lower probability of survival. Neonates require a protective environment, adequate nutrition, and special maternal care in order to survive [19]. So, the season of birth, the intake of milk, and the parity–lactation status of the doe all affect the likelihood of survival. The probability of survival at 4 d of age was lower in winter than in spring and summer, when the weight at birth was less than 50 g. If the birth weight is less than the optimum weight, the energy reserves and the thermoregulatory capacity are reduced, and the perinatal mortality increases [20]. If the temperature in the nest is low during their first five days of life, the instantaneous energy production capacity of the young rabbits is insufficient, being unable to compensate for thermal losses through the skin, and the probability of survival decreases [21].

The kits’ fat tissue is high at birth, and decreases thereafter [22]. The ingestion of milk immediately after birth allows the rabbit to save fat tissue, and thus significantly increase its chances of survival [23,24]. The lack of a milk spot at birth increases the mortality of the kits at 4 d of age, irrespective of their birth weight. Similar results were obtained at the first week of age [24,25].

When lactation and gestation were overlapping, the probability of survival was lower than in nulliparous and non-lactating does. It is well known that does undergo a nutritional deficit when lactation and pregnancy overlap [26,27], and that this deficit affects the probability of the kits’ survival.

## 5. Conclusions

The low line leads to a greater uniformity of kit weight at weaning than the high line, although the variability of weight at birth is higher, which could be due to a higher lactation capacity of the doe. In conclusion, selection for litter size variability shows a negative correlated response in the uniformity of weights at birth, and a positive correlated response in survival and the uniformity of weights at weaning, without affecting individual and litter weight.

## Figures and Tables

**Table 1:** Temperature and relative humidity by season.

Season	Temperature (°C)			Relative Humidity (%)		
	Average	Minimum	Maximum	Average	Minimum	Maximum
Winter	14.4	7.5	22.2	63.2	26.6	100
Spring	20.3	15.2	28.6	78.7	43.3	100
Summer	26.9	19.2	33.8	76.9	32.1	100

**Table 2:** Number of kits at birth (number of kits at 4 d of age) by line effect and individual birth weight (g).

	20–34	35–44	45–54	55–64	65–80
Line H	73 (29)	316 (214)	644 (578)	494 (468)	234 (222)
Line L	128 (54)	339 (226)	756 (652)	661 (609)	338 (321)

**Table 3:** General mean, standard deviation (SD), coefficient of variation (CV) for litter size at birth, survival, litter weight, individual weight, and weight distance before weaning.

	Line H			Line L		
	Mean	SD	CV	Mean	SD	CV
Litter size	7.69	2.98	0.38	8.35	2.43	0.29
Survival						
At birth	0.89	0.25	0.28	0.86	0.25	0.29
At 4 days of age	0.88	0.25	0.28	0.87	0.25	0.29
At weaning	0.61	0.34	0.56	0.67	0.34	0.51
Litter weight						
Total at birth (g)	431	118	0.27	450	119	0.26
Live at birth (g)	412	127	0.31	410	128	0.31
At weaning (g)	2518	1183	0.47	2460	1185	0.48
Individual weight						
Live at birth (g)	53.5	11.2	0.21	54.0	11.2	0.21
Dead at birth (g)	46.3	11.2	0.24	46.0	11.1	0.24
At weaning (g)	493	151	0.31	477	150	0.31
Weight distance						
Live at birth (g)	4.9	4.6	0.94	5.4	4.6	0.85
Dead at birth (g)	6.9	4.6	0.67	6.8	4.6	0.67
Weaned (g)	53.3	45.7	0.86	47.8	46.4	0.97

**Table 4:** Features of the marginal posterior distribution of the differences between the high and the low litter size variability lines for litter size at birth, survival, litter weight, individual weight, and weight distance before weaning.

	H	L	H-L	HPD_95%_		P
Litter size						
At birth	7.7	8.3	−0.6	−1.1;	−0.2	1.00
Survival						
At birth	0.89	0.87	0.02	−0.03;	0.06	0.79
At 4 days of age	0.88	0.87	0.01	−0.04;	0.05	0.67
At weaning	0.62	0.67	−0.05	−0.12;	0.01	0.93
Litter weight						
Total at birth (g)	440	443	−3	−15;	8	0.73
Live at birth (g)	409	411	−2	−14;	9	0.65
At weaning (g)	2461	2404	57	−84;	205	0.78
Individual weight						
Live at birth (g)	53.5	54.1	−0.4	−1.7;	0.8	0.75
Dead at birth (g)	46.3	46.1	−0.2	−2.4;	1.9	0.60
At weaning (g)	495	480	15	−17;	47	0.82
Weight distance						
Live at birth (g)	4.9	5.4	−0.5	−0.9;	0.0	0.97
Dead at birth (g)	7.0	6.8	0.2	−0.9;	1.3	0.68
Weaned (g)	54.1	47.8	6.3	0.2;	12.3	0.98

H = median of the high line; L = median of the low line; H-L = median of the difference between the high and the low lines; HPD_95%_ = highest posterior density region at 95%; P = probability of the difference being ˃0 when H-L ˃ 0, and probability of the difference being <0 when H-L < 0.
